# Cholesterol-Lowering Effect of Beta Glucan Extracted from *Saccharomyces cerevisiae* in Rats

**DOI:** 10.3797/scipharm.ISP.2015.07

**Published:** 2016-02-14

**Authors:** Kusmiati, F. X. Rizky Dhewantara

**Affiliations:** 1 Research Center for Biotechnology – LIPI, Jl Raya Bogor Km 46, Cibinong Bogor 16911, Indonesia; 2 Faculty of Pharmacy – Univ. of Pancasila, Srengseng Sawah Jagakarsa, Jakarta 12640, Indonesia

**Keywords:** Beta glucan, *S. cerevisiae*, Cholesterol, Peroxidase lipid

## Abstract

Glucans are present in fungi, plants, algae, and bacteria. β-Glucan, one of the major cell wall components of *Saccharomyces cerevisiae*, has been found to enhance immune functions. Glucans are glucose polymers with an α- or β-type glycosidic chain. The role of (1→3)-β-D-glucan is in the maintenance of yeast cell wall shape and rigidity. Studies reveal that soluble glucans can lower total cholesterol and LDL levels in patients with hypercholesterolemia. The important benefit of β-glucan is to improve the immune system and to decrease cholesterol levels in the blood. Several studies have reported the benefits of β-glucan as: antiseptic, antioxidant, anti-aging, immune system activators, protection against radiation, anti-inflammatory, anti-diabetic, anti-cholesterol etc. In this research *S. cerevisiae* was cultured in yeast extract–peptone–glucose (YPG) broth medium to produce beta-glucan. Cells were harvested at the stationary phase, washed, and disrupted by means of sonication method. The obtained cell walls were used to prepare alkali-soluble β-glucan (glucan-S_1_). In this regard, 2% sodium hydroxide (NaOH) and 3% acetic acid were used in alkaline–acid extraction, respectively.

Potential use of beta-glucan extract as an anticholesterol agent was tested using *Sprague dawley* strain rats. The experiments were divided into eight groups with four replicates: Group I (normal control), group II (fed with cholesterol without beta-glucan), group III (fed with cholesterol + atorvastatin), group IV (fed with cholesterol + β-glucan standard), group V–VIII (fed of cholesterol + β-glucan of *S. cerevisiae* with each dose of 10, 20, 30, and 40 mg / BW. Rats were fed with cholesterol for 14 days, except for group I. Analysis of blood was carried out to determine total cholesterol, triglycerides, and malondialdehyde.

The results showed that beta-glucan crude obtained from *S. cerevisiae* cultures was 6.890g.L^−1^. Βeta-glucan extract of *S. cerevisiae* can reduce total cholesterol approaching normal values at doses of 10 mg of 32.79 % (blood plasma) and 33.71 % (in the liver). The extract was capable of reducing triglyceride levels in a dose of 10 mg of beta-glucan 64.43 % (blood plasma) and at a dose 30 mg of beta-glucan 19.45 % (liver). Beta-glucan treatment at a dose of 40 mg can reduce MDA levels of 45.22 % (blood plasma) and 42.64 % (liver).

## Introduction

Cholesterol is classified into the class of lipid (fat) containing steroid alcohol components. Cholesterol is synthesized in the liver and becomes essential in forming bile salt and steroid hormones such as testosterone in males and progesterone and estrogen in women. In addition, it is also essential in forming vitamin D and becomes a source of energy [1]. Cholesterol is not easily absorbed by the body. The substance comes into the organs of the body through the lymphatic system.

Cholesterol can be derived from foods of animal origin, meat, eggs, dairy, fisheries, brain tissue, nerve tissue, and egg yolk [2]. At normal levels, cholesterol serves to keep the body healthy. Unbalanced cholesterol level or the one exceeding normal limits (> 200 mg / dL) can cause the formation of atherosclerotic plaque which is harmful to health [3]. Atherosclerosis is a blockage of the arteries due to the buildup of cholesterol in the arterial wall. If the atherosclerosis happens in the arteries that supply oxygen to the heart, it can lead to coronary heart disease (CHD). Coronary heart disease is the leading cause of death in many countries including Indonesia [4].

Alternative medication for lowering cholesterol levels in the body can involve the use of the β-glucan, i.e. polysaccharide derived from wheat, seaweed, fungi, and yeast. It is reported that *S. cerevisiae* is a type of yeast that can synthesize β-glucan in the cell wall. The structure of the cell wall of *S. cerevisiae* contains mannan, chitin, and polysaccharide types of β-1,3-glucan and β-1,6-glucan which serves to strengthen the cell structure and becomes food reserve [5]. Glucose molecules in the β-glucan are linked with β-glycosidic bonds forming the polymer main chain. β-Glucan from *S. cerevisiae* culture was obtained through the extraction process using acid-base neutralization method. β-glucan can function to improve the process of phagocytosis and interleukin formation in humans and animals. Both processes play an important role in the body’s defense system. β-glucan is also capable of lowering blood cholesterol levels. Results of other studies report the benefits of β-glucan as an antiseptic, antioxidant, anti-aging, protection against radiation, anti-inflammatory, antidiabetic, and so on [6]. The role of β-glucan in the food industry has been popular since 1989, i.e. as food stabilizers and flavorings. β-Glucan has been marketed in Taiwan, Korea and Japan. Even, in Japan β-Glucan has been made into a synthetic meat for vegetarians.

This research examined the potential of crude β-glucans extracted from cultures of *S. cerevisiae* as anti-cholesterol. Administration of β-glucan is done orally in various doses to *S. dawley* rats that experienced hyperlipidemia. Parameters measured include total cholesterol, triglycerides, and malondialdehyde levels in the blood and liver. As a comparison, Atorvastatin as blood cholesterol-lowering drug commonly used by the people was used. Enzymes that work on atorvastatin is the enzyme HMG-CoA reductase inhibitors and will work from the liver to regulate LDL receptors on hepatocytes, so it can lower plasma total cholesterol [4].

## Results and Discussion

### Dry weight of β-Glucan Crude from culture of S. cerevisiae

The amount of β-Glucan crude resulted from culture of *S. cerevisiae* is 6890.42 mg/L. The result is the average of three repetitions. β-Glucan Crude was further tested in vivo to lower the cholesterol levels in *S. dawley* rats that experienced hypercholesterolemia.

### Total Cholesterol Levels In Blood and liver of S. dawley Rats

Cholesterol in the body is useful as material used for hormones synthesis and membrane or cell-wall formation. Eighty percent of cholesterol is formed in the liver and the rest comes from various types of food and beverages. However, excessive levels of cholesterol in the body can increase the risk of various diseases. In this study, the effort of increasing cholesterol concentration can be done by feeding high cholesterol mixed with PTU, so the condition of hypercholesterolemia is achieved. Cholesterol high-diets in can raise rats’ blood cholesterol level. The use of goat fat and cooking oil along with the butter can lead to increased level of cholesterol in the blood because the materials contain saturated fatty acids. Saturated fatty acids cause the formation of smaller VLDL particles containing more cholesterol. In addition PTU, antithyroid, can inhibit the formation of thyroid hormones. Then, the thyroid inhibition will increase the blood cholesterol concentration through endogenous cholesterol biosynthesis [11]

The treatment of β- glucan crude extract against *S. dawley* rats which was aimed to prevent hypercholesterolemia was conducted after the rats passed the adaptation period. The effect of β-glucan treatment can be determined by comparing the cholesterol levels in the treatment group to negative group (without the treatment of β-glucan crude). Standard β-Glucan is used as a positive control. It was aimed to determine the potential of anti-cholesterol β-glucans crude extracted from *S. cerevisiae* compared to the standard β-glucan. In addition, Atorvastatin, a compound – naphtyl ester of butyric acid, was used as a positive control. Mechanism of atorvastatin was in in forms of 3-hydroxy-s3-methyl-glutaryl-coenzyme A (HMG-CoA) reductase that functions as a catalyst in the formation of cholesterol. HMG-CoA reductase was responsible for reducing of cholesterol synthesis and increasing the number of receptors of Low Density Lipoprotein (LDL) contained in the liver cell membrane. It caused many LDL missing in plasma. Atorvastatin tended to raise HDL cholesterol [12, 13].

Decrease in total cholesterol levels in the plasma and liver of *S. dawley* rats were determined by the enzymatic method using cholesterol kit. Reaction occurred, the enzyme cholesterol esterase, hydrolyzed ester cholesterol into free cholesterol and fatty acids. Cholesterol oxidase enzyme oxidized free cholesterol into kolestenon and peroxide hydrogen. Further, peroxide hydrogen reacted with 4-aminoantipirin and phenol to form complex red quinoeimine. The absorbance of red color formed was measured with uv - vis spectrophotometer at 500 nm.

**Fig. 1:**
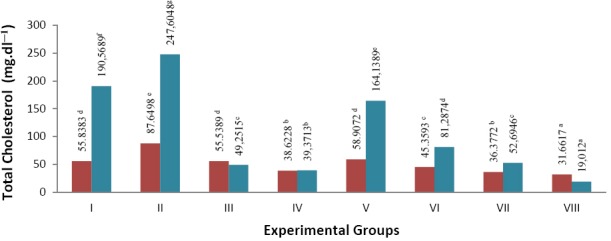
Note: numbers followed by the same letters are not significantly different Total cholesterol levels in blood plasma and liver of hypercholesterolemia *S. dawley* rats (mg.dl^−1^)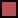 blood plasma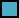 liver

Effect of β-glucan crude extract from culture of *S. cerevisiae* on the decrease of total cholesterol in the blood and liver of *S. dawley* rats can be seen in Figure 1.

In the treatment group II (negative control), in which animals were not given β-glucan crude extract to neutralize cholesterol, there was an increase in blood plasma cholesterol levels (56.97%) and liver (29.93%) compared to group I, i.e. the normal group without β-glucan extract treatment and were not fed cholesterol. The most significant decrease of cholesterol levels was shown in the group VIII (animals were fed with cholesterol and treatment of β-glucan extract 40 mg): reduction of blood total cholesterol was 63.88 % and reduction of cholesterol in the liver was 92.32 % compared to group II (negative control). The treatment of β-glucan extract 10mg (group V) showed that cholesterol level was approaching normal cholesterol levels (group I). A decline of up to 32.79 % was obtained in blood plasma and 33.71 % in the total cholesterol to the liver of group II (negative control). It was shown that treatment of β-glucan crude extract suppressed the cholesterol levels in the blood and liver of rats.

Distribution of data normality test results in total cholesterol levels in the blood plasma and liver of *S. dawley* rats indicated that the data were normally distributed in each treatment group, so it was proceed with the one-way ANOVA. Results of one-way ANOVA for total cholesterol levels in the blood plasma and liver significance of data showed P (0,000) < 0.05, which showed that treatment of β-glucan significantly affected blood plasma total cholesterol levels and liver (Table 1 and 2). Test was followed by Duncan’s multiple range test (DMRT) which showed that there were significant differences between the treatment groups.

**Tab. 1:**
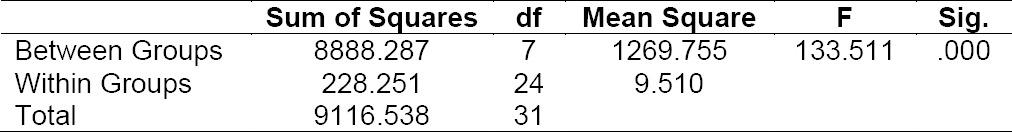
ANOVA analysis of total cholesterol levels in blood plasma of *S. dawley* rats

**Tab. 2:**
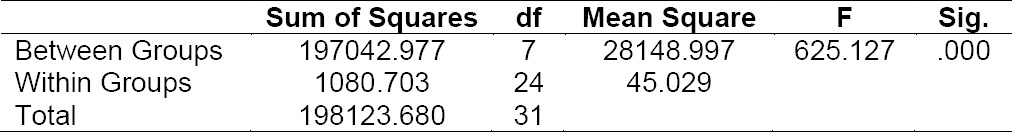
ANOVA analysis of total cholesterol levels of *S. dawley* rats’ liver

### Triglyceride Levels in Blood and Heart of S. dawley Rats

Triglycerides are lipids having ester structure, which is composed of three molecules of free fatty acids and a glycerol molecule. Triglycerides are a type of fat found in the blood and is the result of decomposition of foods containing fat and cholesterol that has been consumed and comes into the body. After experiencing a process in the body, triglycerides will be absorbed by the intestines and comes into the blood plasma, then it is distributed throughout the body in the form of chylomicrons and Very Low Density Lipoprotein (VLDL). As VLDL, triglycerides are formed by the liver with the help of insulin from the body. Meanwhile, triglyceride which is outside the liver and is in certain tissue such as tissue of blood vessels, fat tissue will be hydrolyzed by the enzyme lipoprotein lipase. Hydrolysis residue will be metabolized into LDL cholesterol.

In this study, the determination of triglyceride levels was done by enzymatic methods. The reaction occurring in the determination of plasma and liver triglyceride levels was the formation of a complex compound 4-(p-benzokinon-monoimino)-fenazon having brownish yellow color, the absorbance of which was then measured at 500 nm. The mechanism of the reaction was triglycerides with lipoprotein lipase enzyme was hydrolyzed into glycerol and fatty acids. Glycerol, with the presence of adenosine triphosphate (ATP) by the enzyme glycerol kinase, was converted into glycerol-3-phosphate. Furthermore, glycerol-3-phosphate was oxidized by glycerol phosphate oxidase enzyme into dihydrodiasetonphosphate and hydrogen peroxide. Hydrogen peroxide formed reacted with 4-aminofenazon and 4-chlorophenol forming compound 4-(p-benzoqinone-monoamino)-fenazon having brownish yellow color [14].

**Fig. 2:**
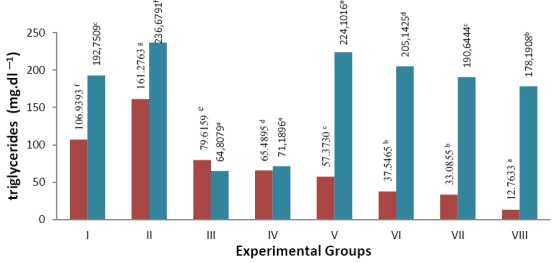
Note: numbers followed by the same letter are not significantly different Triglycerides level in the blood plasma and liver of hypercholesterolemia *S. dawley* rats (mg.dl^−1^)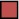 blood plasma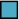 liver

Effect of β-glucan crude extract from cultures of *S. cerevisiae* toward the reduction of triglycerides in the blood and liver of *S. dawley* rats that experienced hyper-cholesterolemia can be seen in Figure 2.

Results of measurements of blood plasma triglyceride levels showed an increase in group II (negative group), which is the treatment group that was not given β-glucan crude extract compared to group I (normal). There was an increase in blood plasma triglyceride levels (50.81%) and liver (22.79%). The most significant triglyceride levels decrease was shown in the group VIII (β-glucan extract 40 mg): blood plasma triglyceride levels reduced by 92.09% and could reduce liver triglyceride levels by 24.71% against negative control. Decreased levels of triglycerides in the liver was more significant in group III (positive atorvastatin) of 72.62% and group IV (positive β-glucan standard) at 69.92%. Triglyceride levels approaching normal values in blood plasma was obtained in group V (treatment of β-glucan extract 10mg) at 64.43% and of the liver at 19,45% was obtained group VII (β-glucan extract 30mg). The results could indicate that the addition of β-glucan crude extract can reduce levels of triglycerides in *S. dawley* rats that experienced hypercholesterolemia.

Data normality distribution test results of triglyceride levels in blood plasma indicates the data are normally distributed in each experimental group, while the levels of triglycerides in the liver showed that the data were not normally distributed in each treatment group. Data processing of triglycerides levels in the blood plasma can be followed by one-way ANOVA test. Results of ANOVA showed significance of P (0,000) <0.05; there is real difference in giving treatment to the levels of triglycerides in the blood plasma (Table 3). Testing continued with Duncan’s multiple range test test (DMRT): the levels of triglycerides in the blood plasma showed significant difference in each treatment group. Testing levels of triglycerides in the liver followed by non-parametric test using the Kruskal-Wallis test. Results showed the data of significance P (0,000) <0.05: the treatment showed significant effect on the levels of triglycerides in the liver. Testing continued with Mann-Whitney test: there was a real difference in each treatment group to the levels of triglycerides in the liver.

**Tab. 3:**
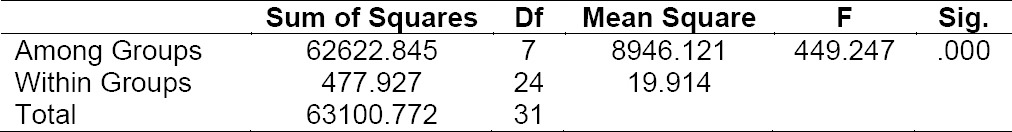
ANOVA analysis of triglyceride levels of blood plasma *S. dawley* rats

### MDA in Blood and Liver of S. dawley Rats

The presence of free radicals in the body can be characterized by the presence of lipid peroxide, which reacts to produce secondary products which form malondialdehyde (MDA). Measurement of lipid peroxides can be done indirectly by measuring the level of MDA. Method was done by heating using thiobarbiturate acid reagent to form a pink color. Measurement of plasma and liver MDA levels with a spectrophotometer were conducted using methods Thiobarbituric Acid Reactive Substances (TBARS); was performed on λ 532 nm [15]. Measurable results can describe the levels of lipid peroxides. Tetraethoxypropane compound (TEP) was used in constructing standard raw curve as it can be oxidized in acid condition and resulted aldehyde compounds that can react with TBA. High levels of free radicals in the body could be indicated by high levels of MDA in plasma. Cholesterol and PTU became source of free radicals. Hence, the addition of both cause elevated levels of MDA in group II (negative control). The group was not given β-glucan crude extract to neutralize the cholesterol feed given orally.

**Fig. 3:**
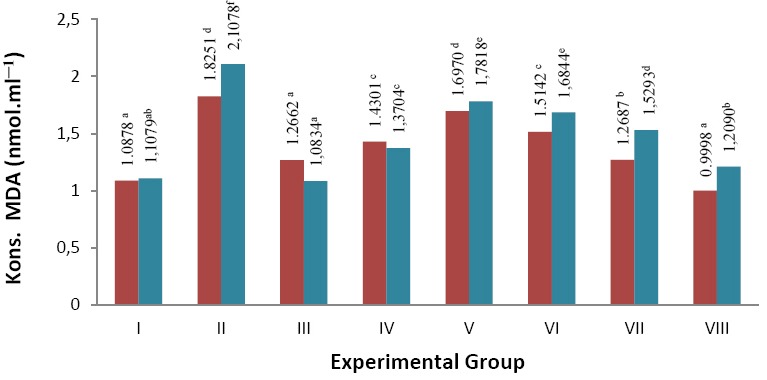
Note: numbers followed by the same letter are not significantly different Blood plasma 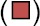 and liver 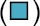 Malondialdehida (MDA) levels of *S. dawley* white rat that have hypercholesterolemia (nmol.ml^−1^).

Effect of β-glucan crude extract from culture of *S. cerevisiae* toward decrease of MDA in blood and liver *S. dawley* rats can be seen in Figure 3.

Results of measurements of blood plasma and liver MDA of *S. dawley* rats showed an increase respectively 67.81 % and 90.25% is in group II (negative control) which was the experimental group without any treatment of β-glucan crude extract compared to group I (normal control). It was due to the high content of cholesterol derived from feed given and the induction of PTU. The treatment of β-glucan 40 mg (group VIII) was able to reduce levels of MDA in blood plasma of 45.22 % and liver of 42.64 % compared to group II (negative control). Positive control treatment (atorvastatin) was able to reduce levels of MDA in blood plasma 30.62 % and liver 48.60% compared to the negative control. Meanwhile, the positive control (β-glucans) lowered blood plasma MDA levels up to 21.64 % and liver 34.98%. The results of this study indicates that the addition of β-glucan crude extract MDA can reduce levels of the liver caused by high cholesterol feed.

Analysis of the data normality distribution of blood plasma and liver MDA levels indicated that the data was normally distributed. Then, the data were analyzed by one-way ANOVA. MDA levels in blood plasma and liver showed data results of significance P (0,000) < 0.05, which indicated that the anti-cholesterol treatment significantly affected the MDA (Tables 4 and 5). Furthermore, It was tested that DMRT showed noticeable differences among the treatment groups.

**Tab. 4:**
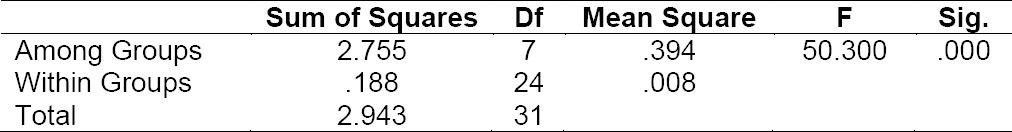
ANOVA analysis of MDA levels of Blood plasma *S. dawley* rats

**Tab. 5:**
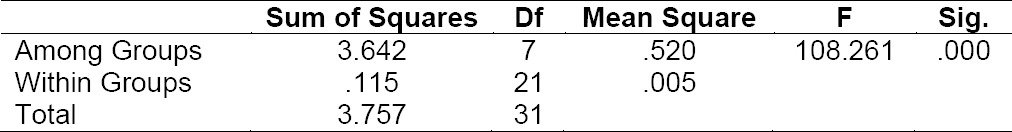
ANOVA analysis of MDA level of *S. dawley* rats’ liver

In general, there are increased total cholesterol, triglycerides, and MDA levels in *S. dawley* rats that were fed with additional high cholesterol. Extra food given was animal fat 10%, egg yolk 5%, cholesterol 1%, PTU 0.01%, and t-BHP0.5 mM. Increasing cholesterol levels in rats was performed exogenous and endogenous. Exogenously, the increase was performed by treating animal fat, egg yolk, and cholesterol; while PTU was used to endogenously increase cholesterol levels and t-BHP is a strong oxidizer/organic peroxides which are easily decomposed and in the reaction form free radicals. PTU is tireo-statistical drug that can inhibit the release of a hormone secreted from the thyroid gland and is a thionamid group. Treating these foods simultaneously is expected to provide a synergistic effect. From the results of the data analysis can it be concluded that the greater the dose of β-glucan crude extract, the greater its effect on the decrease in total cholesterol, triglycerides, and MDA levels of *S. dawley* rats experiencing oxidative stress due to high cholesterol feed plus PTU and t-BHP.

## Experimental

### Microorganism

*S. cerevisiae* was obtained from the collection of Research Center for Biotechnology-LIPI, isolated from commercial yeast of SAF (Jakarta, Indonesia).

### Animals

*Sparague Dawley* rats weighing 200–250 g, purchased from the Faculty of Veterinary Medicine, Bogor University of Agriculture, Indonesia were caged individually under a 12:12 h light: dark cycle (light on at 6:00 h) and controlled room temperature (22±2°C) with cubes of standard rodent diet and water for 6 days before the experimentation. All experiments were performed according to a protocol approved by a local animal care ethical committee.

### Preculture of S. cerevisiae.

Two loops of *S. cerevisiae* cultures were inoculated into 20 ml YPG liquid medium with composition (w / v) of 1% yeast extract, 2% peptone and 2% glucose. The cultures were incubated in a shaker incubator at 30°C for 48 hours at a speed of 150 rpm.

### Production and Extraction of Beta Glucan [7, 8]

A total of 2% of *S. cerevisiae* precultures was inoculated into 150 ml YPG liquid medium. The cultures were incubated for 5 days in a shaker incubator at 30ºC with a speed of 150 rpm. In the stationary phase, cultures were extracted to obtain β-glucan by centrifuging it at 6000 rpm for 15 minutes, then the supernatant was discarded. Biomass was hydrolyzed with 0.75 M sodium hydroxide for 6 hours at a temperature of 75°C. The solution was centrifuged at a speed of 6000 rpm at room temperature for 15 minutes, then the supernatant was discarded. Biomass was added with 15 ml of 0.5 M acetic acid, then heated at a temperature of 50°C, and then centrifuged at a speed of 6000 rpm at a temperature of 15°C for 20 minutes, then the supernatant was discarded, followed by washing for three times. Biomass was then rinsed with water and centrifuged at a speed of 6000 rpm for 15 minutes, then the supernatant was discarded. The rinsing/flushing was done twice. Biomass was added with ethanol then centrifuged at a speed of 6000 rpm for 15 minutes, the biomass obtained was a crude β-glucan, and then dried at a temperature of 50ºC and weighed (g).

### Beta Glucan as an Anti-cholesterol Tested on S. dawley Rats (in vivo).

32 rats were adapted to the environment and standardly fed for a week. The rats were made hypercholesterolemia by adding high cholesterol feed for 14 days, the rats put in group I were excluded. Experimental rats were divided into 8 groups with 4 replications treatment, namely:

**Table:**
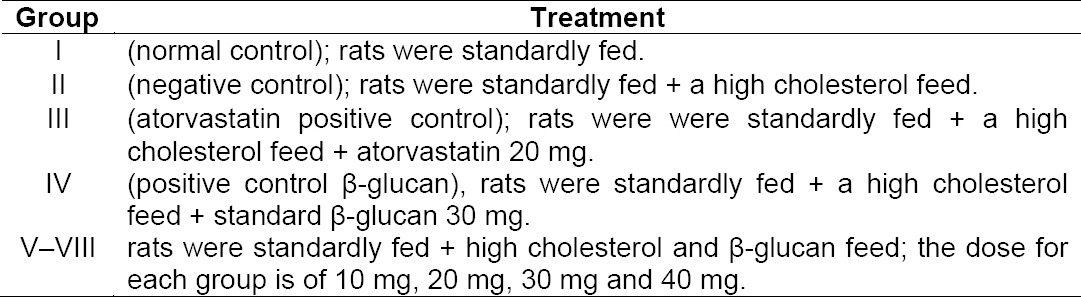


### Preparation of blood samples

Before blood sampling, rats were fasted for 18 hours and then weighed (g). An amount of 3 up to 4 ml of blood was taken from the heart, collected into a test tube containing an anticoagulant, remained for a few momments, and then centrifuged at a speed of 3000 rpm for 10 minutes. Cholesterol, triglycerides, and MDA of the resulting supernatant were measured. 50μl blood plasma diluted with distilled water ad 1000 mL then was homogenized.

### Preparation of liver sample

A total of 1 g liver sample was pulverized, 0.9% NaCl solution was added and then inserted into the Eppendrof tube. Plasma was separated from the liver cells by centrifuging for 10 minutes at a speed of 10,000 rpm.

### Analysis of total cholesterol in the blood and liver [9]

Measurement of total cholesterol was carried out by *colorimeter enzymatic CHOD PAP test* method. A pipette 100 µL of blood and liver plasma was added by 100 mL cholesterol kit reagent. Tubes containing samples were incubated for 5 minutes at a temperature of 370C. Serum samples were measured using a spectrophotometer with a wavelength of 500 nm. The same test was carried out for the blank (cholesterol kit reagent) and standard solution (standard cholesterol of 200 mg.dL^−1^).

Total cholesterol level is calculated by the following formula:


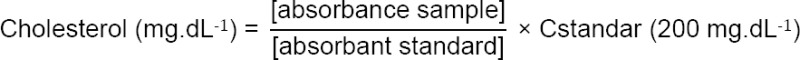


### Analysis of triglyceride in blood and liver [9]

Triglyceride levels measurements was carried out using CHOD PAP colorimeter enzymatic test method. Pipette 100 mL of blood and liver plasma was added by triglycerides kit reagent, and then was incubated for 5 minutes at a temperature of 37°C. Serum samples were measured using a spectrophotometer with a wavelength of 500 nm. The same test was carried out for the blank (triglycerides kit reagent) and standard solution (standard triglycerides 200 mg.dL^−1^).

### Analysis of malondialdehyde (MDA) [10]

#### Standard curve

Each of 10.0; 20.0; 40.0; 60.0; and 80.0 µL of tetraethoxy propane (TEP) solution (1: 80000) was added with 250 mL H2O, 1.25 mL of 20% trichloroacetat acid (TCA), and 0.5 mL of 0.67% tiobarbiturat acid (TBA) and then homogenized. The mixture was heated for 30 minute in boiling water, then rapidly cooled. The absorption was measured by spectrophotometer at λ 532 nm. Concentrations of MDA were calculated from a standard curve prepared from 1,1,3,3 tetraethoxypropane and stated as nmol / ml for the plasma.

Each of these levels of standard solution of TEP and absorbance standard curve was plotted as TEP and then regression line was calculated,


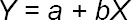


Where: a = y intercept, b = slope, y = absorption at a wavelength of 532 nm, x = Conc. of TEP (nmol/mL).

with correlation coefficients (r) to determine the corelation between concentration and standard uptake reference.

#### MDA concentration

As much as 250 µL of blood plasma and supernatant of liver was treated similarly as the TEP standard and absorption was measured at λ 532 nm. MDA levels were calculated using standard curve regression equation TEP.


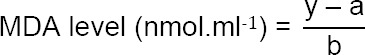


Where: y = absorption at a wavelength of 532 nm, a = y intercept, b = slope

## Conclusion

Experimental results can be summarized as follows:

1. The average crude β-glucan produced by culture of *S. cerevisiae* was 6890.42 mg.L^−1^.
2. β-glucan crude extract can lower total cholesterol levels approaching normal values in blood plasma and liver of *S. dawley* rats was obtained in group V (treatment of β-glucan extract 10mg), triglyceride levels in blood plasma was obtained in group V (treatment of β-glucan extract 10mg) and liver was obtained in group VII ((treatment of β-glucan extract 30mg) and MDA levels in blood plasma and liver was obtained in group VIII (treatment of β-glucan extract 40mg).
